# Aspergillosis and pulmonary tuberculosis co-infection in a 9-year-old with B-cell acute lymphoblastic leukemia

**DOI:** 10.1093/omcr/omad082

**Published:** 2023-08-20

**Authors:** Wirapatra Iamwat, Krit Cheawcharnprapan, Patcha Yenjabog, Oranooj Lertkovit, Daranee Isaranimitkul, Thiraporn Kanjanaphan

**Affiliations:** Division of Pediatric Pulmonary and Critical Care, Department of Pediatrics, Faculty of Medicine, Vajira Hospital, Navamindradhiraj University, Bangkok, Thailand; Division of Pediatric Neurology, Department of Pediatrics, Faculty of Medicine, Vajira Hospital, Navamindradhiraj University, Bangkok, Thailand; Division of Pediatric Critical Care, Department of Pediatrics, Faculty of Medicine, Vajira Hospital, Navamindradhiraj University, Bangkok, Thailand; Division of Pediatric Hematology and Oncology, Department of Pediatrics, Faculty of Medicine, Vajira Hospital, Navamindradhiraj University, Bangkok, Thailand; Division of Pediatric Hematology and Oncology, Department of Pediatrics, Faculty of Medicine, Vajira Hospital, Navamindradhiraj University, Bangkok, Thailand; Division of Pediatric Infectious Disease, Department of Pediatrics, Faculty of Medicine, Vajira Hospital, Navamindradhiraj University, Bangkok, Thailand

## Abstract

This case report highlights the infrequent occurrence of coinfection involving invasive aspergillosis and *Mycobacterium tuberculosis* (MTB) in pediatric patients. We present the case of a 9-year-old Thai girl diagnosed with B-cell acute lymphoblastic leukemia, who experienced prolonged febrile neutropenia lasting 1 month during chemotherapy. Chest computed tomography (CT) revealed lung nodules with an air crescent sign, while CT angiography of the brain detected an infected ruptured brain aneurysm, which exhibited septate hyphae with acute angle branching, consistent with invasive aspergillosis. Despite voriconazole treatment, the patient’s high-grade fever and dyspnea persisted. Further investigations revealed a lung abscess and wedge resection confirmed AFB 1+ and positive MTB detection via polymerase chain reaction, leading to the initiation of combined treatment for pulmonary tuberculosis and invasive aspergillosis. Considering drug–drug interactions was an essential aspect of the management. This case report highlights challenges of coinfection between invasive aspergillosis and MTB.

## INTRODUCTION

Invasive aspergillosis is a serious *Aspergillus* infection [1] that commonly affects immunocompromised hosts [1]. *Mycobacterium tuberculosis* (MTB), which is characterized by hemoptysis, chest pain, weight loss, fever and night sweat, is a leading cause of mortality; however, this disease is particularly difficult to diagnose among children [2]. Both invasive aspergillosis and MTB are serious and life-threatening infections. Unfortunately, their manifestations are similar, thereby difficult to distinguish. Chronic pulmonary aspergillosis secondary to pulmonary TB and coinfection between these two diseases occur in adults, resulting in subacute invasive aspergillosis, especially among those with mild-to-moderate immunosuppression [3–5]. However, the prevalence of such coinfection remains unclear and has been rarely described in the pediatric population.

## CASE REPORT

A 9-year-old girl, born in Thailand, was diagnosed with B-cell acute lymphoblastic leukemia (B-cell ALL) and presented with epistaxis. She underwent chemotherapy as per the very-high-risk ALL protocol, including vincristine, doxorubicin, L-asparaginase, prednisolone and intrathecal methotrexate. Before starting chemotherapy, her chest X-ray was normal, and tuberculin skin test reported 0 mm. However, she failed to respond to the induction phase and required augmented-consolidation treatment with cyclophosphamide, high-dose cytarabine, mercaptopurine and intrathecal methotrexate.

**Figure 1:**
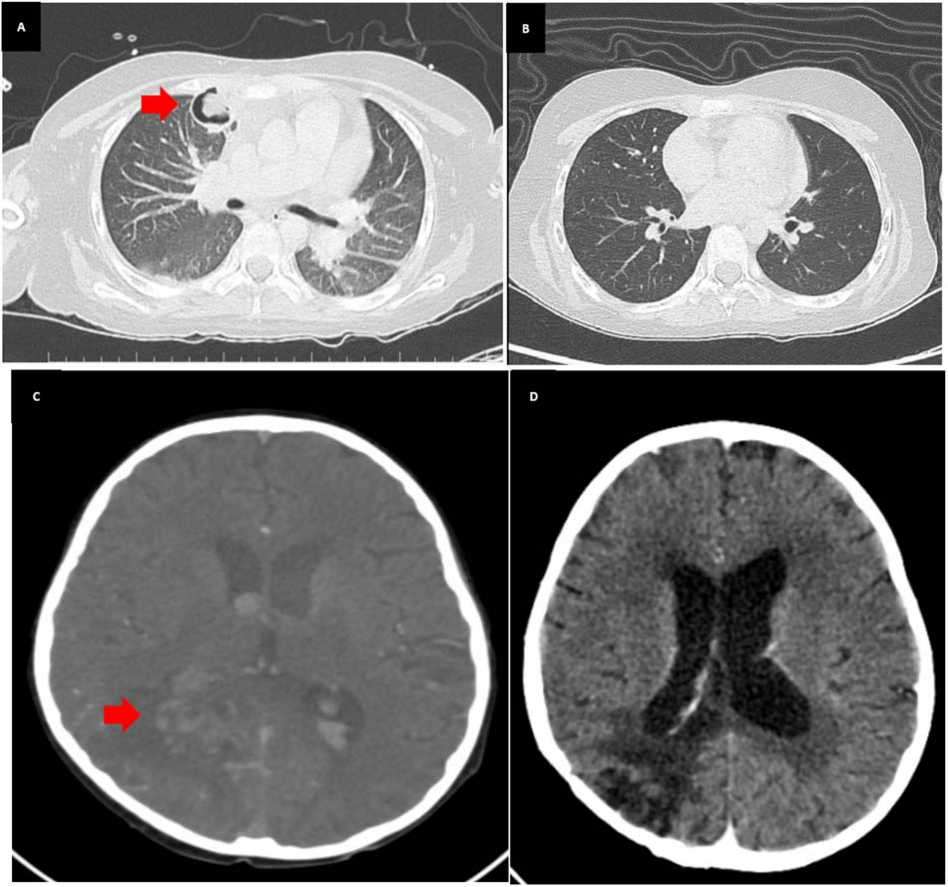
CT chest and CT brain before and after treatment in this patient. (**A**) Contrast-enhanced CT of the chest detected an air crescent sign at the anterior segment of the right upper lobe of the lungs. (**B**) Absence of air crescent sign after treatment. (**C**) CT angiography of the brain revealed an outpouching lesion at the right posterior cerebral arteries. (**D**) After treatment and follow-up, the ruptured aneurysm and intraventricular hemorrhage improved.

After a week of augmented-consolidation treatment, the patient developed febrile neutropenia, characterized by a complete absence of neutrophils. Despite receiving IV piperacillin/tazobactam for 4 days, she continued to experience prolonged febrile neutropenia for duration 4 days. Hemoculture, urine culture and serum Aspergillus galactomannan results were reported as negative. To investigate further, a computed tomography (CT) chest scan was performed, revealing an air crescent sign at the anterior segment of the right upper lobe (2.7 × 2.2 × 2.3 cm^3^) and multiple nodules in the lungs, indicating invasive pulmonary aspergillosis (Fig. 1A).

**Figure 2:**
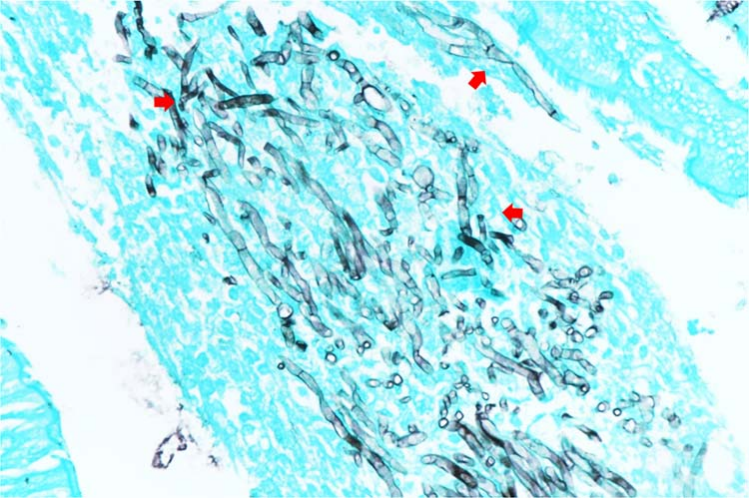
The pathological of brain tissue specimen. GMS stain of brain tissue revealed thin septate dichotomous branching hyphae.

She was started on intravenous voriconazole, the recommended treatment for invasive aspergillosis. However, after 2 days of therapy, she developed a progressive headache on the right side, along with neck stiffness. At this point, the patient’s antibiotic regimen was changed from piperacillin/tazobactam to meropenem. A brain scan identified an outpouching lesion at the right posterior cerebral arteries, suggestive of ruptured infected aneurysms (Fig. 1C). She underwent a surgical procedure to remove the aneurysm, which was confirmed by Gomeri-Methenamine Silver stain (GMS) of tissue biopsy to be caused by Aspergillus (Fig. 2). However, the fungal culture did not yield any growth. Subsequently, the patient was continued on meropenem for 14 days.

**Figure 3:**
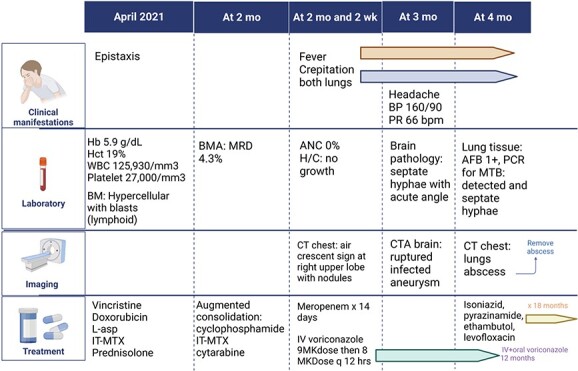
The summary of case presentation. The summary of timeline of clinical manifestations.

During the course of treatment, the patient’s voriconazole trough levels were 2.1 mg/L (8 mg/kg/dose every 12 hours). After treating invasive aspergillosis for 2 weeks, the absolute neutrophil count (ANC) returned to normal. However, she continued to experience prolonged fever and persistent cough for 4 weeks. Bronchoalveolar lavage (BAL) revealed no organisms were detected and negative for galactomannan. A chest CT scan revealed air crescent sign (2.5 × 2.4 × 1.9 cm^3^) at the anterior segment of the right upper lobe. This particular lesion was found to be in contact with the chest wall and had affected the costal cartilage. As a result, the possible diagnoses were considered for lung abscess, specifically caused by aspergillosis, *Staphylococcus aureus*, *Streptococcal pneumoniae* and anaerobic bacteria. Given bacterial considerations, she was placed on Piperacillin/Tazobactam. Surgical intervention in the form of a wedge resection was performed, the resected tissue exhibited caseous necrosis. Gram stain, culture and modified acid-fast bacilli (mAFB) testing of the tissue biopsy yielded negative results. However, the AFB staining revealed a positive 1+, with polymerase chain reaction (PCR) analysis confirming the detection of MTB in the tissue sample. For treatment of Aspergillus, she received IV voriconazole until achieving clinical stability, followed by a transition to oral tablets 400 mg every 12 hours (level was 3.4 mg/L). The co-administration of voriconazole and rifampicin can result in significant drug–drug interactions leading to decreased voriconazole levels and increased rifampicin levels. This interaction has the potential to compromise the effectiveness of voriconazole while also increasing the risk of rifampicin-related side effects. Hence, rifampicin was replaced with levofloxacin, and treatment for both TB and invasive aspergillosis continued. Notably, the patient tolerated medications well. After 3 months, significant improvement was observed in clinical examinations and imaging studies (Fig. 1B and D). The patient’s lesions remained stable, and oral voriconazole was discontinued after 12 months. However, the anti-TB drugs continued for a total of 18 months. The summary of clinical manifestations demonstrates in Fig. 3.

## DISCUSSION

Pulmonary aspergillosis typically presents with symptoms such as fever, hemoptysis, cough and weight loss. An immunocompromised host is a known risk factor for invasive pulmonary aspergillosis [6]. In the case of our patient, who had B-cell ALL and developed febrile neutropenia, and subsequently developed disseminated aspergillosis. However, treating her condition became challenging as her aspergillosis did not respond to voriconazole therapy. Bacterial infection was initially suspected since previous studies reported that 40.3% of patients with aspergillosis also had bacterial coinfection [7]. However, the results of the patient’s hemoculture, urine culture and BAL culture for bacteria were all negative.

Coinfection between MTB and Aspergillus in children is considered rare, primarily affecting patients who are immunocompromised or have previous lung damage [8]. A previous study documented a case of invasive pulmonary aspergillosis and pulmonary tuberculosis in a 48-year-old man with Crohn’s disease who was undergoing infliximab treatment [9]. Furthermore, the tuberculin skin test, sputum and BAL tests for AFB, Gene Xpert MTB/RIF (rifampicin resistance) and culture for *M. tuberculosis* (MTB) all came back negative, and no previous chronic lungs disease, pulmonary TB coinfection was considered less likely. As a result, the patient’s fever and cough persisted, and a chest CT scan revealed a lung abscess, raising suspicion of aspergillosis. However, upon lung biopsy, AFB 1+ was reported, and PCR detected MTB. The similarity of symptoms between pulmonary TB and invasive pulmonary aspergillosis led to misdiagnosis, delayed isolation and delayed treatment. Coinfection between MTB and Aspergillus can occur in immunocompromised children, although it is rare. This case report highlights the challenges in diagnosing and treating coinfections, particularly when drug–drug interactions are involved. It also emphasizes the importance of considering tuberculosis as a differential diagnosis in areas with a high prevalence of contagious tuberculosis, especially when aspergillosis does not respond to standard antifungal therapy.
